# Spontaneous fusion across the apex of severe thoracolumbar Scheuermann’s kyphosis: A surgical consideration

**DOI:** 10.4103/0019-5413.65146

**Published:** 2010

**Authors:** Athanasios I Tsirikos

**Affiliations:** Scottish National Spine Deformity Centre, Royal Hospital for Sick Children, Edinburgh, UK

**Keywords:** Kyphosis, Scheuermann, treatment

## Abstract

Considerable debate exists regarding the pathogenesis, natural history and treatment of Scheuermann’s kyphosis. Surgical correction is indicated in the presence of severe kyphosis which carries the risk of neurological complications, persistent back pain and significant cosmetic deformity. This can be achieved through a posterior-only or an anteroposterior approach. Spontaneous fusion in association with Scheuermann’s kyphosis has not been previously described. This is an important consideration if surgical correction of the kyphosis is planned. Two patients with severe thoracolumbar Scheuermann’s kyphosis who developed spontaneous posterior and anteroposterior fusion across the apex of the deformity are presented. The surgical treatment and final outcome is discussed.

## INTRODUCTION

Scheuermann^1^ (1920) described a rigid kyphosis of the thoracic or thoracolumbar spine, which is the most common cause of structural kyphosis and thoracic back pain in adolescence. The condition is characterized by an acutely angular kyphosis with the apex in the thoracic or thoracolumbar spine associated with reduced anterior vertebral growth resulting in vertebral body wedging, endplate irregularities, and early disc degeneration.

Surgical treatment in adolescents with Scheuermann’s kyphosis (SK) is considered when there is progressive, severe deformity more than 70-80°, which cannot be controlled with bracing, as well as in the presence of disabling pain resistant to conservative measures using non-steroidal anti-inflammatory medication, modification of activities and exercises for a minimum of six months.^2^ Surgical correction might also be considered in patients with severe deformities who express genuine concerns in regard to their appearance.

Biomechanical principles of kyphosis correction include lengthening of the concavity (anterior column) and shortening of the convexity (posterior column) of the deformity. Successful results have been reported by either posterior procedures alone (posterior compression instrumentation and arthrodesis), or combined anterior spinal release and interbody arthrodesis followed by posterior instrumentation and arthrodesis.^3^–^5^

To the author’s knowledge, spontaneous vertebral fusion in association with SK which has not been previously reported. Two patients with very severe thoracolumbar SK who developed spontaneous bony fusion across the apex of the deformity are presented and their treatment, as well as surgical outcome is discussed.

## CASE REPORTS

### Case 1

A male patient aged 17 years and two months presented with a severe thoracolumbar SK. He was otherwise healthy but markedly overweight with a body mass index (BMI) of 37. There was no history of spinal infections or injuries and no skeletal dysplasias. There were no associated medical co-morbidities. He had been previously followed but not treated in another spinal unit since the age of 16 years during which period his kyphosis gradually progressed and became symptomatic. On presentation in our clinic, the patient complained of persistent back pain located in the thoracolumbar junction, which affected the level of his activities. The pain did not radiate to his legs and he had no neurological symptoms.

On clinical examination, he had a sharply angular kyphosis of 105° with the apex in the thoracolumbar junction, as well as bilateral hamstring tightness. Neurological examination was normal. There was an associated thoracolumbar scoliosis measuring 30° and no evidence of spondylolysis or spondylolisthesis. The lateral radiograph of the spine showed bridging osteophytes anteriorly across the apex of the kyphosis. Magnetic resonance imaging (MRI) of the spine was performed when he was initially seen at the age of 16 years and showed spinal cord attenuation across the thoracolumbar junction but no cord signal change, no disc herniation and no intraspinal anomalies. There was also no evidence of the bridging osteophytes on the MRI which indicates that these developed spontaneously in order to stabilize the spine as the kyphosis progressed further and the disc spaces collapsed anteriorly. The MRI did not report fusion of the posterior elements across the levels of the kyphosis, as well as congenital vertebral abnormalities.

Indications for surgery included severe back pain refractory to conservative measures and modification of activities, as well as the extreme degree of deformity and risk for further progression possibly causing neurological complications.

The patient underwent kyphosis correction at the age of 17 years and 11 months when the deformity measured 115° and only corrected to 100° on a supine hyperextension radiograph against the bolster. The surgery involved a combined single-stage anterior and posterior spinal arthrodesis extending from T4 to L3 with the use of posterior pedicle hook/screw/rod instrumentation and autologous rib bone graft. The anterior approach was performed through a right thoracotomy on the convexity of the scoliosis. The diaphragm was retracted distally but not divided in order to provide access to L1. During the anterior stage, the anterior longitudinal ligament was found to be ossified from T10 to L1 with bridging osteophytes extending circumferentially from T11 to T12 at the apex of the kyphosis and displacing the major vessels anteriorly. The intervertebral discs from T9 to T12 were very stenotic and immobile. The osteophytes were excised both on the convexity and concavity of the associated thoracolumbar scoliosis. The anterior longitudinal ligament was released and complete discectomies back to the posterior longitudinal ligament were performed from T7 to L1.

**Figure 1:**
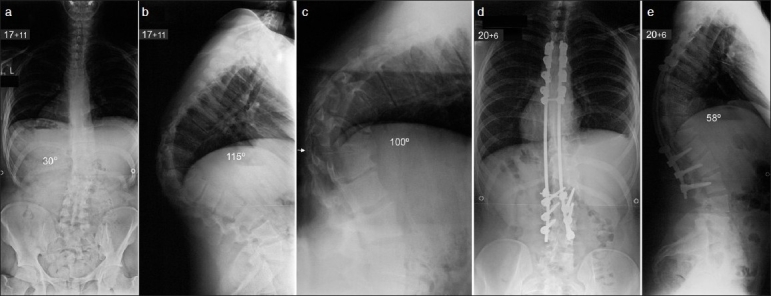
Preoperative posteroanterior radiograph (a) of Patient 1 shows a mild right thoracolumbar scoliosis measuring 30°. Lateral radiograph (b) shows a severe thoracolumbar kyphosis measuring 115°. Preoperative supine lateral radiograph in hyperextension against the bolster (white arrow, (c) shows the kyphosis correct to only 100°. Posteroanterior (d) and lateral (e) radiographs at 2.6-year-follow-up show normal coronal balance of the spine and a residual kyphosis of 58°

During the posterior exposure, the spine was found to be spontaneously fused across the apex of the kyphosis from T9 to L1. There was no evidence of congenital vertebral anomalies. Extensive posterior apical closing wedge osteotomies were performed from T7 to T12. The fused facets and ossified ligamentum flavum were excised and the spine was mobilized at completion of the anterior and posterior osteotomies. The kyphosis was corrected using a cantilever maneuver with the rods simultaneously attached from proximal to distal under intraoperative spinal cord monitoring recording motor evoked potentials (MEPs), as well as cortical and cervical somatosensory evoked potentials (SSEPs). The spinal cord monitoring signals remained stable throughout the procedure. An excellent correction of the kyphosis to 58° was achieved [Figure 1]. Autologous rib graft harvested during the anterior stage of the procedure was used to enhance a bony fusion across the levels of the instrumentation.

The patient had an uncomplicated postoperative course and mobilized following application of a spinal brace to provide additional support. He was discharged 18 days after surgery. The brace remained for four months and the patient gradually returned to normal activities including sports six months after surgery. At latest follow-up, 2.6 years following kyphosis correction, the patient had no complaints of his back; he had normal activities and a BMI of 26. Radiographs of the spine showed no evidence of pseudarthrosis and no loss of kyphosis correction or junctional deformity either proximal or distal to the instrumentation.

### Case 2

A girl aged 12 years was diagnosed with a thoracolumbar kyphosis by her family doctor and was initially followed by physiotherapists to provide exercises for her abdominal and dorsal muscles. She was not referred for an orthopedic assessment until the age of 17 years and eight months when she presented in our clinic with a severe thoracolumbar SK. She was otherwise healthy but overweight with a BMI of 38. There was no history of spinal infections or injuries and no skeletal dysplasias. At presentation, the patient had complaints of persistent back pain in the thoracolumbar junction, which restricted her activities. She had no complaints of leg pain and no neurological symptoms.

On clinical examination, there were no neurological abnormalities and the spine demonstrated an extreme kyphosis (113°) with the apex in the thoracolumbar junction, as well as a sharply angular gibbus. She had a mild left thoracolumbar scoliosis of 15° and no spondylolysis or spondylolisthesis. There was no radiographic evidence of anterior bony fusion across the apex of the kyphosis and no bridging osteophytes. An MRI scan excluded intraspinal anomalies, and showed small disc bulges from T8 to T11 with normal spinal cord signal. It also showed no evidence of congenital vertebral anomalies.

The indications for surgery included chronic thoracolumbar back pain, as well as the extreme degree of deformity and potential for neurological complications.

**Figure 2:**
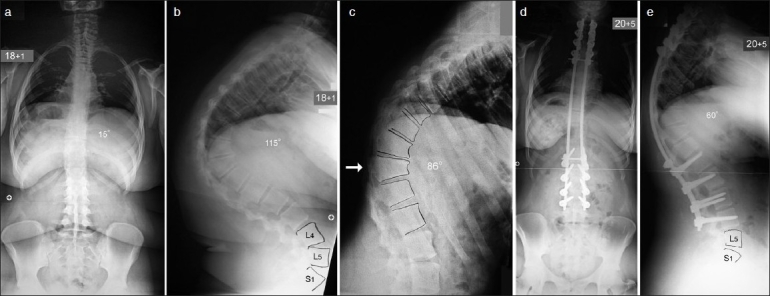
Preoperative posteroanterior radiograph (a) of Patient 2 shows a mild left thoracolumbar scoliosis of 15°. Lateral radiograph (b) shows a severe thoracolumbar kyphosis of 115°. Preoperative supine lateral view in hyperextension against the bolster (white arrow, (c) shows the kyphosis correct to 86o. Posteroanterior (d) and lateral (e) radiographs at 2.3-year-follow-up show no scoliosis and a residual kyphosis of 60°

The patient underwent kyphosis correction at the age of 18 years and one month when the deformity measured 115° and only corrected to 86° on supine hyperextension radiograph against the bolster. The surgery involved a single-stage posterior spinal arthrodesis extending from T2 to L4 with the use of posterior pedicle hook/screw/rod instrumentation and autologous iliac crest bone graft. During the posterior exposure, the spine was found to be spontaneously fused across the apex of the kyphosis from T9 to L1 with fused facet joints and an ossified ligamentum flavum. There was no evidence of congenital vertebral anomalies. Extensive posterior apical closing wedge osteotomies were performed from T6 to T12. The fused facets and ossified ligamentum flavum were excised and the spine was mobilized at completion of the osteotomies. The kyphosis was corrected using a cantilever maneuver with the rods simultaneously attached from proximal to distal under intraoperative spinal cord monitoring recording MEPs, as well as cortical and cervical SSEPs. The intraoperative spinal cord monitoring remained stable throughout the procedure. An excellent correction of the kyphosis to 60° was achieved [Figure 2]. Autologous iliac crest graft was used to achieve a solid bony fusion across the levels of the instrumentation.

The patient made a good recovery and mobilized following application of a spinal brace. She was discharged 20 days after surgery. The brace remained for four months and the patient progressively returned to normal activities seven months after surgery. At latest follow-up, 2.3 years following kyphosis correction, the patient had no complaints of her back; she had normal activities and a BMI of 28. Radiographs showed no evidence of pseudarthrosis and no recurrence of the kyphosis or junctional deformity proximal or distal to the instrumented fusion.

## DISCUSSION

Scheuermann’s disease is considered a benign condition resulting often in small deformity and limited symptomatology with most of the complaints settling at skeletal maturity. Murray *et al*.,^6^ performed a natural history and long-term follow-up study and showed that patients with SK adapted well to their condition with few functional limitations. However, in the presence of severe kyphosis (>75°) there may be progressive deformity and debilitating back pain.^3^–^5^,^7^ Degenerative spondylosis and disc herniations can develop into adulthood primarily across the apex of the kyphosis, causing more significant back pain and/or neurological compromise.^8^–^11^ The thoracolumbar type of Scheuermann’s disease is more likely to result in a painful progressive kyphosis in adult life if left untreated compared to the thoracic.

The decision for surgical treatment should be based primarily on the severity of patients’ symptoms and self-perception, and secondarily on the degree of kyphotic deformity. If deformity is the primary concern of the patient, the benefit of correcting it should not be underestimated. The indications for kyphosis correction in our two patients included severe thoracolumbar pain refractory to conservative measures and modification of activities, patients’ dissatisfaction regarding their cosmetic deformity, as well as the extreme degree of kyphosis and possible risk for spinal cord compression.

Spontaneous fusion in association with SK has not been reported. The development of bony ankylosis may represent the natural history of an extreme deformity as an attempt of the spine to auto-stabilise. A combination of factors including a rigid deformity, which limits significantly active movement of the spine, as well as anterior vertebral body wedging with severe adjacent disc stenosis which induces bridging osteophyte formation may result in the development of spontaneous fusion across the apex of the kyphosis either posteriorly or anteroposteriorly.

In both patients the preoperative MRI assessed the intraspinal structures but did not recognise the solid fusion across the posterior bony elements at the apex of the kyphosis. A computed tomography (CT) scan with 3D reconstruction would have illustrated the bony anatomy at the apex of the kyphosis giving valuable information to assist surgical planning. This is recommended in the presence of rigid thoracolumbar SK which does not correct in hyperextension, especially if the plain radiograph shows anterior bridging osteophytes.

The posterior fusion across the thoracolumbar junction in both patients, as well as the ossification of the anterior longitudinal ligament in Patient 1 was only identified intraoperatively. Resection of the anterior longitudinal ligament and bridging osteophytes along with complete discectomies was required to mobilise anteriorly the apical levels of the kyphosis in the first patient. The osteophytes extended circumferentially across the anterior aspect of the vertebral bodies and the major vessels had to be displaced and protected during osteophyte excision. Both patients required resection of the ossified ligamentum flavum, spinous processes and opposing margins of the laminae followed by posterior closing wedge osteotomies including the fused superior and inferior facets across the apical levels in order to allow for kyphosis correction. This surgical strategy achieved excellent correction of the deformity which was maintained at last follow-up.

The role of anterior spinal release is more important in severe and rigid SK; however, the addition of an anterior procedure to the posterior instrumented fusion increases significantly the risk of complications. In the author’s experience,^2^ an anterior release is not required if the kyphosis corrects to approximately 80-90° on a lateral hypextension radiograph with the patient supine against a bolster as occurred in Patient 2. In this patient, the isolated spontaneous posterior fusion was addressed with extensive posterior osteotomies. In Patient 1, an anterior procedure was performed due to the rigidity of the kyphosis which only corrected to 100° on the hyperextension radiograph. The presence of an ossified anterior longitudinal ligament, as well as bridging osteophytes across the intervertebral discs at the apex of the kyphosis limit the ability to correct the deformity through a posterior-only procedure and should be considered indications for an additional anterior spinal release.

In both patients, the posterior instrumented arthrodesis extended from the most proximal level of the measured kyphosis to include the first lordotic segment distally as determined on the standing lateral radiograph and this prevented the development of junctional kyphosis at either end of the fusion. A hybrid construct with pedicle hooks proximally and pedicle screws distally was used to secure spinal fixation above and below the levels of the posterior closing wedge osteotomies. In the author’s experience, the use of a hybrid system without hook claw configuration proximally and no supralaminar hooks allows intact interspinous ligament and facet joints between the most cephalad instrumented vertebra and the one above, thus, reducing the risk of junctional kyphosis in the upper thoracic spine.

In conclusion, spontaneous posterior or anteroposterior fusion can occur across the apex of severe thoracolumbar SK; this should be taken into account when surgical correction is anticipated. Preoperative CT imaging can recognize the abnormal anatomy which includes ossification of the anterior longitudinal ligament and ligamentum flavum, development of anterior bridging osteophytes, as well as ankylosis of the facet joints at the levels of the acute kyphosis. In the presence of an isolated posterior fusion, segmental posterior closing wedge osteotomies with complete excision of the ossified ligamentum flavum and fused facets should mobilise the thoracolumbar spine and allow for kyphosis correction. An additional anterior spinal release including complete discectomies, resection of the anterior longitudinal ligament and osteophytes is required if the bony fusion extends anteroposteriorly.
